# Management of avascular necrosis of femoral head at pre-collapse stage

**DOI:** 10.4103/0019-5413.45318

**Published:** 2009

**Authors:** Ramesh Kumar Sen

**Affiliations:** Department of Orthopaedics, PGIMER Chandigarh, India

**Keywords:** Avascular necrosis, core decompression, fibular grafting, osteonecrosis

## Abstract

In osteonecrosis the success of interventions that forestall or prevent femoral head collapse and maintain hip function would represent a substantial achievement in the treatment of this disease. A review of recent literature regarding bisphosphonate, anticoagulant, and vasodilators and biophysical modalities have demonstrated efficacy in reducing pain and delaying disease progression in early stage osteonecrosis. Though it has been considered still insufficient, to support their routine use in the treatment or prevention of osteonecrosis of the hip. Core decompression with modification of technique is still one of the safest and most commonly employed procedures with evidence based success in the pre-collapse stage of AVN of femoral head. The additional use of bone morphogenic protein, and bone marrow stem cells may provide the opportunity to enhance the results of core decompression. At present, the use of large vascularised cortical grafts, the other surgical procedure with high success rate is still not common due to technical difficulty in surgery. Likewise osteotomies are also not getting common as arthroplasty is getting more acceptable, so is awaited without any intermediate big surgical interventions.

## INTRODUCTION

The avascular necrosis (AVN) or osteonecrosis of the femur head (ONFH), a disease with many etiological factors, affects young population and if not managed timely, leads to the collapse of femur head eventually requiring hip arthroplasty. Early presentation of avascular necrosis of femur head may be painless; however the ultimate presentation is painful limitation of hip motion.^1^ Passive movements of hip are also restricted. There is a high chance of bilateral presentation. Careful clinical history is important to find any of the risk factors. The Harris hip score is one of the most common clinical scales used for assessing the hip status.

The antero-posterior radiographs of the affected hip show the principal area of AVN. However, because the anterior and posterior acetabular margins overlap the superior portion of the femoral head, subtle abnormalities in the subchondral region may be missed. So good quality lateral X-rays of the femoral head are very important. A cross table lateral radiograph is less satisfactory than a frog leg lateral to reveal the architectural details of the femoral head.^2^ Technetium 99m diphosphonate imaging (bone scanning) is a useful technique for detecting osteonecrosis.^3^ Multiple studies have demonstrated that MRI is the most accurate of all imaging modalities.^2^,^4^ Double line signal on T2-weighted image is virtually pathognomic for AVN. Also, the single density line, which is so often seen outlining the necrotic lesion on the T1 weighted image is thought to be highly specific for AVN. MRI can also show the re-vascularization front and gives objective evidence of tissue changes in response to treatment allowing sequential evaluation of AVN lesions on follow-up.^5^ In comparison Computerized tomography (CT) scanning, is useful only in separating the late pre-collapse stages of AVN from the early collapse stage.^2^

Ficat described a four stage (I through IV) classification system, which is based on standard radiographs.^6^ In Stage I the radiographs are normal. In Stage II the contour of the femoral head is normal but the radiographs show evidence of bone remodeling including cystic and sclerotic areas. Stage III involves flattening of the femoral head. In Stage IV, there is joint space narrowing with secondary degenerative changes in the acetabulum. Steinberg *et al*,^7^ expanded the Ficat system by dividing Stage III lesions into femoral heads with and without collapse or hips with acetabular involvement. The Japanese Investigation Committee introduced the concept of location of the lesions.^8^ Association Research Circulation Osseous (ARCO) proposed a new international classification in 2001 using MRI.^9^ This system uses the four part staging system of Ficat, while adding the extent of involvement advocated by Steinberg^7^ and the location of involvement proposed by the Japanese Investigation Committee.^8^

While patients with advanced AVN usually end up with hip arthroplasty, some of those with early diagnosis of the lesion (at pre-collapse stage) have been managed with hip salvage surgery. Newer modalities including variety of drugs have also been used for non-operative management of AVN. It is thus considered worthwhile to have a review of therapeutic modalities of AVN femoral head before the lesion reaches the stage when arthroplasty becomes an inevitable option.

## MATERIALS AND METHODS

A Medline search (1966 to 30^th^ November 2008) was performed to identify the all randomized, clinical trials published in English language journals on the treatment of early stage AVN [Figure 1]. Studies with advanced stage AVN were excluded. The search string consisted of the terms “AVN, ON, non-operative treatment, core decompression, vascular graft, osteotomy”. The searches were limited to “English” in the language field and “Human” in the study type field. Additional studies were identified from review articles and articles cited in original papers. Abstracts, letters to the editor, and unpublished data were not considered. Related Cochrane reviews and systemic reviews were searched for additional references. In total 80 studies were identified which met the inclusion criteria

**Figure 1A:**
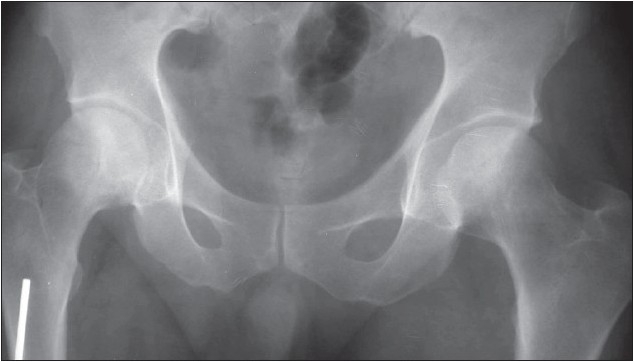
Anteroposterior radiograph of pelvis. No lucency, sclerosis or subchondral collapse noted in the femoral head. Joint space is preserved.

**Figure 1B:**
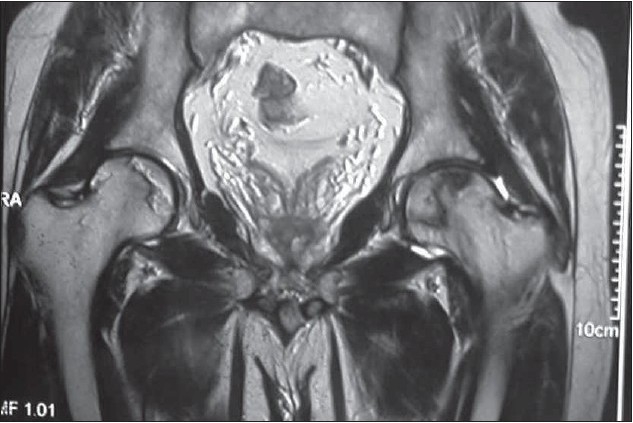
T2 weighted coronal section shows avascular necrosis involving bilateral femoral heads with reparative border appearing hypo/hyperintense (double rim sign) on right side indicating granulation and sclerosis respectively. Dark line bordering the AVN focus on left side represents sclerosis. The cartilage and acetabulum are normal.

**Figure 1C:**
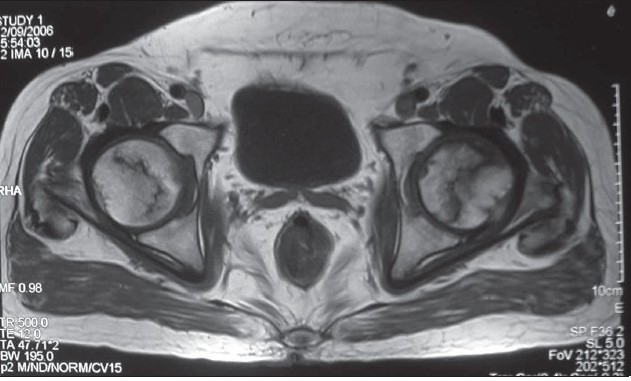
Axial T1 weighted image shows fatty high signal within the necrotic area and hypointense line around them due to reparative new bone.

## RESULTS

The medline search for term osteonecrosis provides 4912 references. With limits to English language there are 3228 total articles with 228 review articles. Adding the limits in the search for ‘Humans, Clinical Trial, Meta-Analysis, Practice Guideline, Randomized Controlled Trial, Review, English’, the number is reduced to 326 including 224 review articles. Excluding ‘review articles’ there are just 104 articles in the limits set by ‘Humans, Clinical Trial, Meta-Analysis, Randomized Controlled Trial, English’, out of these only 53 deal with outcome non arthroplasty studies. The observations in these 53 studies and linked review articles have explained the factors in outcome in this avascular necrosis femur head.

## NON-OPERATIVE TREATMENT OPTIONS

### Restricted weight-bearing

Reduced weight-bearing on the affected hip joint have been advised with the expectation of preventing femoral head collapse during the healing of osteonecrotic bone. There are only 5 studies with reference to weight bearing as treatment modality. Small pre-collapse lesions have been observed to have a more favourable prognosis, taking much longer to become symptomatic and may even resolve spontaneously.^10^ The factors that appear to be related to resolution are early, asymptomatic disease (ARCO stage I) and small lesion size (a modified index of necrotic extent of < 25). Hernigou *et al*,^11^ have observed that clinical and radiographic signs of the disease in asymptomatic hips with a very small asymptomatic lesion progress more slowly than do signs in hips with a large symptomatic stage-II lesion. In their study,^11^ among the lesions that had a volume of < 5 cm^3^ involving < 10% of the volume of the femoral head, by ten years 88% had become symptomatic and 73% eventually collapsed. Thus they stressed the need for prolonged follow-up of even the smallest lesions.

Among these there is a meta-analysis of non-operative methods of twenty-one studies encompassing 819 hips, which revealed that with restricted weight bearing modality, only 22.7% of the hips were satisfactory clinically (range 0 to 44%).^12^ Seventy-six per cent of the hips (range 44 to 100%) required hip arthroplasty or a salvage procedure. It has been now been universally agreed that conservative treatment of osteonecrosis of the femoral head by restriction of weight bearing is not appropriate.

### Pharmacological agents

The use of lipid-lowering drugs, anticoagulants, vasodilators, and bisphosphonates have been considered on the basis of specific physiological risk factors for osteonecrosis such as lipid emboli, adipocyte hypertrophy, venous thrombosis, increased intraosseous pressure, and resorption of bone.

### Lipid lowering agents

The Pubmed search with the limits of avascular necrosis and lipid lowering drugs shows 3 papers. Wang *et al*,^13^ had reported the ability of lovastatin to prevent corticosteroid-induced adipogenesis and osteonecrosis of the femoral head. It has been suggested that in systemic lupus erythematosus, osteonecrosis of the femoral head may develop as a result of a physiological diversion of mesenchymal stem cells toward an adipocytic lineage. So treatment with lovastatin is likely to prevent this diversion of normal osteoblastic cellular differentiation. It has also been observed that both the pro-inflammatory and the pro-coagulant properties of the vascular endothelium that are induced by antiphospholipid antibodies can be controlled by treatment with Statins. Pritchett^14^ in a clinical study reported that, at a mean of 7.5 years (minimum, five years), osteonecrosis of the femoral head had developed in only three (1%) of 284 patients who were taking high-dose corticosteroids along with statin drugs (lipid clearing agents that dramatically reduce lipid levels) in contrast to prevalence of 3-20% reported for patients receiving high-dose corticosteroids without statins.^15^

Glueck *et al*,^16^ reported the use of anabolic steroid stanozolol (6 mg/day) to treat patients who had hypofibrinolysis associated with a high level of plasminogen activator activity and one patient who had a high level of lipoprotein in the serum. All patients showed a decrease of symptoms at one year following treatment.

The effects of the prostacyclin derivative iloprost, used as a vasodilator, have been studied by Meizer *et al*,^17^ in patients with osteonecrosis of the femoral head and bone marrow edema syndrome. Among seventeen patients with early-stage osteonecrosis of the femoral head, all had clinical and radiographic improvements at one year after treatment with this agent.

Glueck *et al*,^18^ used enoxaparin (60 mg/day for twelve weeks) to treat patients who had thrombophilic or hypofibrinolytic disorders and early stages of osteonecrosis of the femoral head. At two years, thirty-one (89%) of thirty-five hips had not required surgery and were still in the Ficat and Arlet Stage-I or Stage-II disease as assessed radiographically. They suggested that treatment of the underlying coagulation disorders may arrest or delay the progression of the osteonecrosis and anticoagulation with warfarin or LMW heparin, if started in the early stages of lesion before collapse, may also result in improvement of the condition.

8 clinical reports of the use of alendronate in osteonecrosis highlight the potential benefit of alendronate for patients with osteonecrosis of the femoral head.^19^,^20^ At an average of one year (range, three months to five years) after treatment of sixty patients (100 hips) with this agent (10 mg/day), Agarwala *et al*,^19^ found clinical improvement, with a reduction in patient disability scores and only six patients (ten hips) requiring surgery. Similar findings have been reported by Lai *et al*,^20^ in a study of forty patients with Steinberg Stage-II or III osteonecrosis of the femoral head who were either treated with alendronate (70 mg/day for twenty-five weeks) or assigned to a control group. At a minimum of twenty-four months, only two of the twenty-nine hips in the alendronate group had loss of femoral head integrity compared with nineteen of the twenty-five hips in the control group (*p*< 0.001).One hip in the alendronate group and sixteen hips in the control group underwent total hip arthroplasty (*p*< 0.001). However, the doses required and duration of therapy is yet to be clearly established. The long-term effect of these drugs, many of which have an extremely long half-life, on normal bone homeostasis and turnover also merits caution.

### External, biophysical, nonoperative modalities

Pulsed electromagnetic field stimulation, is reported to be useful for treatment of osteonecrosis in 4 reports.^21^–^24^ This is reported in one retrospective analysis as a treatment method for AVN^21^ in addition to case reports and review articles. The rationale for the use of pulsed electromagnetic fields in the treatment of osteonecrosis of the femoral head rests on two fundamental mechanisms of action: (1) pulsed electromagnetic fields can play an important role in the local control of inflammation and (2) pulsed electromagnetic fields favour the repair activity that has been observed in osteonecrotic areas of the femoral head and can potentiate the healing process by stimulating neovascularisation and new bone formation.^22^

Extracorporeal shockwave therapy has been utilized in Europe for treatment of early-stage disease. There are only 2 papers in Pubmed and the only study is by Wang *et al*,^23^ who compared the results of such therapy in twenty-three patients (twenty-nine hips) with the results in a group treated with non-vascularized fibular grafting. At a mean of twenty-five months, 79% of the shock-wave group had improved Harris hip scores compared with 29% of the group treated with non-vascularized fibular grafting.

The use of hyperbaric oxygen (HBO) has been made in AVN with the perception that HBO improves oxygenation, reduces oedema, and induces angioneogenesis; thus causing a reduction in intra osseous pressure and improvement in microcirculation. Apart from few case reports and review articles there is only one English study in Pubmed, by Reis *et al*,^24^ involving 16 hips in 12 patients, all with Steinberg stage 1 disease, gave each patient 100 consecutive days of HBO, which involved breathing 100% oxygen via a mask at 2-2.4 atmospheres pressure for 90 minutes. Though the follow-up period is poorly defined they reported that 13 of the 16 femoral heads subsequently appeared normal on MRI after this treatment.

## OPERATIVE TREATMENT OPTIONS

The surgical interventions have been performed much more commonly in avascular necrosis of femur head with the intention of preserving the joint, and in advanced lesions for joint replacement procedures.

### Core decompression

There are 15 retrospective and prospective studies in English language for Core decompression of the femoral head as an early treatment of avascular necrosis. Ficat and Arlet^25^ reported 1.7 per cent good or very good results on core decompression in Stage-I and in Stage-Il avascular necrosis at an average follow-up of 7.9 years. Hungerford^26^ reported similar results. The rationale for the use of core decompression is based on the concept that increased intra-medullary pressure is involved in the pathogenesis of avascular necrosis. The aim of the proponents of core decompression is to decrease the intramedullary pressure and thus arrest or reverse the process of avascular necrosis before it is evident radiographically.^27^

Wang *et al*,^28^ have shown short-term effect of increased femoral-head blood flow due to core decompression in the rabbit model. The results of study showed that the decrease in femoral head blood flow due to prolonged steroid therapy was reversed by core decompression. Femoral head perfusion can return to a normal or slightly elevated state, four weeks after treatment. Core decompression is expected to relieve the pain and to allow creeping substitution to the necrotic area by bringing the blood supply through the drill channels.

Chan *et al*,^29^ retrospectively evaluated MRI of 32 hips with avascular necrosis of the femoral head before and after the core decompression and bone grafting. Most lesions that appeared stable on MRI were clinically also stable or improved. It was also concluded that MRI can be used to demonstrate changes in size and signal characteristics as well as femoral head collapse after core decompression and bone grafting.

A meta-analysis^30^ of 24 reports analyzing 1,206 hips treated by core decompression with or without cancellous bone grafting revealed an overall clinical success rate of 63.5% (range 33 to 95%). Less than 33% of the hips required a replacement or salvage procedure during the follow-up period.

Technique of Core decompression: The conventional method of doing core decompression involves the use of an 8–10 mm trephine or cannula inserted under fluoroscopic guidance to penetrate the lesion. Complications can occur with multiple drillings with the use of these large-diameter trephines which can weaken the femoral head or when the trephine penetrates the femoral head, can injure the articular cartilage, and enter the joint space.^31^ In addition, if the core tract is started in the subtrochanteric or diaphyseal area, rather than entering through the metaphyseal region of the proximal femur, the stress risers created can lead to a subtrochanteric fracture.^31^ A change in this technique of core decompression has been suggested lately with the claim of being safer.^32^,^33^ The procedure of core decompression by multiple small drillings was presented in the annual ARCO meeting by Kim *et al*,^32^ in 2004. They compared the results of the efficacy of two decompressive methods (multiple drilling vs conventional core decompression) for the treatment of pre-collapse osteonecrosis of the femoral head in a consecutive series of 54 patients. They reported that radiographically and clinically, high failure was significantly related to the larger size and laterally located lesion in both groups. The average pre-operative and the last HHS was 86.7 to 73.7 in single core decompression and 87.0 to 74.6 in multiple drilling. The group who had undergone multiple drilling had significantly longer time before collapse (mean 42.3 months vs 22.6 months, *p* =0.011) and lower rate of collapse within 3 years after operation (55.0% vs 85.7%, *p* =0.03) than single large core decompression.

Mont *et al*,^33^ in 2004 reported similar results with the use of small multiple drillings (3-mm drill bits) to do core decompression. Postoperatively, their patients were maintained at approximately 50% weight bearing for 5–6 weeks using a cane or crutch in the opposite hand from the hip that was operated. If the patient had bilateral core decompression, two crutches were used for a 4-point gait. After 5–6 weeks, the patients were advanced to full weight bearing as tolerated. High-impact loading such as jogging and jumping was not permitted for 12 months. Rehabilitation throughout recovery to include hip abductor strengthening and ROM exercises was encouraged. If patients were asymptomatic at 10–12 months postoperatively with no radiographic evidence of collapse, they were allowed to resume all usual activities, including higher impact loading activities (such as running). It was also observed that this procedure compared favorably to historical complication rates for core decompression that often occurred 10–15% of the time and included femoral fracture or head blowout.^34^ In this series, there were no serious complications that would be expected as an advantage of using 3.2-mm Steinman pins percutaneously to do the procedure. They opined that this was an appropriate method of core decompression when treating symptomatic patients with small-sized or medium-sized precollapse lesions.

## USE OF OSTEOINDUCTIVE SUBSTANCES ALONG WITH CORE DECOMPRESSION

### Bone morphogenic proteins

In one of the studies to evaluate the efficacy of bone morphogenetic protein enhanced bone graft in preventing disease progression in osteonecrosis of the femoral head, Mont *et al*,^35^ used a modified trapdoor technique and bone morphogenetic protein enriched bone graft substitute through a window at the femoral head-neck junction in 23 patients. They reported successful clinical results (a Harris hip score of 80 points or greater and no additional procedures) in 18 of 21 hips (86%) at a minimum follow up of 36 months (mean, 48 months; range, 36–55 months). However, this procedure required an extensive dissection, and it was also technically more difficult than a standard core decompression. Lieberman *et al*,^36^ reviewed the results of 15 patients treated with core decompression in conjunction with an allo-implant composite of allogeneic, antigen-extracted, autolyzed cortical bone perfused with human bone morphogenetic protein and non-collagenous proteins. Radiographic progression of disease was prevented in 14 of 17 hips at an average of 53 months (range, 26–94 months). Only one of 15 hips, that were classified as Ficat Stage II-A developed collapse. The other two hips that progressed already had collapse of the femoral head before the procedure. The data suggested that core decompression may be more effective if combined with osteoinductive and/or angiogenic factors.

### Bone marrow mesenchymal cell grafting

Mesenchymal stem cells (MSC) from adult bone marrow are multipotent that can differentiate into fibroblastic, osteogenic, myogenic, adipogenic and reticular cells.^37^ These cells may also provide a potential therapy for bone repair.

Osteonecrosis is associated with a decrease in progenitor cells in the proximal femur.^38^,^39^ Normally, in the adult, haematopoietic marrow is absent in the femoral head but red marrow persists in the proximal shaft of the femur.^40^ MRI studies have indicated that the conversion of red to fatty marrow occurs prematurely in some patients with avascular necrosis at the upper end of the femur.^39^ As a consequence, intramedullary vascularity is altered and this may be a predisposing factor for osteonecrosis. The lack of osteogenic cells can also influence the bone repair which occurs after osteonecrosis. A decrease in osteogenic stem cells in the femoral head has been documented beneath the sequestrum and in the inter-trochanteric region.^41^ With the addition of autologous bone marrow along with core decompression, better results have been obtained.^42^–^44^ This procedure of autologous stem cell transplantation has been standardized with the guidelines that these should be instilled in concentration of 2X10^6^ stem cells in non-traumatic pre-collapse stage of avascular necrosis femur head.^45^

Gangji *et al*,^46^ (2005) performed a randomized control study, where 10 patients were given bone marrow stem cells in addition to core decompression (CD), along with 8 control cases where only CD was performed. It was observed that in a period of 24 weeks, level of pain was significantly decreased from 37.8+8.4 mm at baseline to 18.5±6.2 mm at 6 months (*p* =0.016) compared to no significant decrease in control group even uptill 24 months. The Lequesne index in stem cell group decreased from 7.7±1.5 to 3.0±1.1 at 6 months. The WOMAC score also reduced from 30±5 to 18±7 at 6 months. Subsequently all these changes progressed accordingly in subsequent follow up until 24 months. But in the control group of patients, the similar improvement was not observed neither at 6 months nor subsequently. Yan *et al*,^47^ (2006) again reported the clinical efficacy and safety of the treatment of osteonecrosis of the femoral head by percutaneous decompression and autologous bone marrow mononuclear cell (BMCs) infusion in a study of 44 hips in 28 patients. Deltro *et al*,^48^ (2008) published their experience in 8 patients where they assessed the efficacy and safety of autologous bone-marrow mononuclear cells (BMMC) implantation in necrotic lesions of the femoral head in patients with sickle cell disease. After eight months, seven of the eight patients reported improvement from symptoms. There was a significant postoperative increase in the HHS (98.3 ± 2.5 points) compared to preoperative HHS (78.5 ± 6.2 points) (*p* < 0.001). X-ray evaluation and cell parameters were found to be favorable. They concluded that the autologous bone-marrow mononuclear cells implantation is a safe and effective treatment for early stages of femoral head osteonecrosis in patients with sickle cell disease.

This effectiveness of bone marrow mononuclear cells may be related to the availability of stem cells endowed with osteogenic properties arising from an increase in the supply of such cells to the femoral head through bone-marrow implantation.^45^,^46^ Another possible explanation for the therapeutic effect of bone-marrow implantation is that injected marrow stromal cells secrete angiogenic cytokines, resulting in increased angiogenesis and subsequent improvement in osteogenesis.^49^ The bone marrow also contains the bone morphogenetic proteins such as BMP-2, which are introduced into the femoral head and into the necrotic segment. It has been reported that supplementation of bone marrow stromal cells cultures with FGF-2 resulted in prolonged lifespan of bone marrow stromal cells to more than 70 doublings and maintained their differentiation potential accompanied by an increase of their telomerase size.^49^,^50^

With these developments, such augmentation procedures in core decompression surgery are appearing better options than performing more invasive procedures.

## BONE GRAFTING

Bone grafting has been added to the core decompression in an effort to provide structural support, or to act as scaffolding for repair and allow remodelling of subchondral bone. The procedures can vary from addition of autogenous or allograft cancellous bone to a core decompression, osteochondral grafts, muscle-pedicle bone grafts, free cortical grafts and free vascularized bone grafts with iliac or fibular bone.

Cancellous bone graft harvested from the iliac crest, have been used to fill the defect in the femoral head after complete evacuation of the necrotic bone.^51^–^54^ These bone graft can be introduced through a cortical window in the femoral neck or via a ‘trapdoor’ through the articular cartilage of the femoral head, as first described by Merle d'Aubigne^51^ after dislocating the femur head and exposing the flap from the chondral surface of the femur head. The necrotic segment is removed with curettes and burrs. The void is then filled with iliac crest bone graft with or without graft expanders. This was first performed in conjunction with an osteotomy by Ganz and Buchler.^52^ The term ‘light bulb’ procedure was introduced by Rosenwasser *et al*,^53^ who attempted to perform this procedure of curettage and grafting through neck head junction. In their series 13 of 15 hips, with stage II/III disease, were asymptomatic at a mean of 12 years. Mont *et al*,^54^ had reported their observations with this procedure in 24 Ficat stage III and 6 stage IV hips. With an average follow up of 56 months, 73% their patients had good to excellent results.

Cortical bone strut grafts have been advocated in an effort primarily to provide structural support to the subchondral bone and articular cartilage to prevent collapse during the repair process

### Non-vascularized cortical grafts

Phemister^55^ originally described the technique of using a tibial bone graft. Subsequently with the use of tibial graft placed in the canal after the drilling to provide mechanical support for the articular surface satisfactory results have been reported in 70% of cases.^31^ The search in pubmed showed ten papers of reference to nonvascularized fibular graft. Buckley *et al*,^56^ using fibular grafts reported excellent results in 18 (90%) of 20 hips in which a Ficat and Arlet stage-I or stage-II lesion had been treated by core decompression combined with tibial autografting and fibular grafting (both autogenous and allogenic). It was also suggested that non-vascularised autologous bone grafting has other theoretical advantages i.e. the procedure provides decompression of the avascular lesion and removal of the necrotic bone in order to interrupt the cycle of ischaemia and interosseous hypertension. Grafting of the defect with fresh cancellous bone introduces a scaffold for repair and remodelling of subchondral bone. The use of a non-vascularised graft has been more appealing than that of a vascularised graft because it is less technically demanding and may reduce donor-site morbidity.

However, in a long-term study, Smith *et al*,^57^ observed that with non-vascularized grafts, 40 (71%) of 56 hips had a poor clinical result after a mean follow-up of 14 years (4 to 27). In a recent study, Keizer *et al*,^58^ (2006) described the long-term results of core decompression and placement of a non-vascularised bone graft in the management of avascular necrosis of the femoral head. They treated 80 hips in 65 patients, 18 by a cortical tibial autograft and 62 by a fibular allograft. The mean age of the patients was 36 years. A total of 78 hips were evaluated of which pre-operatively six were Ficat-Arlet stage 0, three stage I, 31 stage IIA, 16 stage IIB, 13 stage III and nine stage IV. A total of 34 hips (44%) were revised at a mean of four years. Survivorship analysis using a clinical end-point showed a survival rate of 59% five years after surgery. They found a significant difference (*p* = 0.002) in survivorship, when using a clinical and radiological end-point, between the two grafts, in favour of the tibial autograft. In their view core decompression, removal of the necrotic tissue and packing of the cancellous grafts into the core track have been the vital parts of the procedure.

There is still no consensus regarding the indications for non-vascularised bone grafting. Proponents recommend it for hips with depression of the femoral head of less than 2 mm.

The use of porous tantalum implants in combination with core decompression has the potential to provide the structural advantages of bone graft without the associated risk of autograft harvest or the infectious complications associated with allograft bone. Veillette *et al*,^59^ prospectively evaluated 54 consecutive patients (60 hips) in whom osteonecrosis of the femoral head was treated with core decompression and insertion of a porous tantalum implant. The associated risk factors included corticosteroid use in 22 patients (26 hips), excessive alcohol consumption in two patients (two hips), none known in 13 patients (15 hips), trauma in six patients (six hips), and other factors in nine patients (nine hips). Nine hips (15.5%) were converted to THA, including six with Stage II disease and three with Stage III disease. Kaplan Meier analysis revealed an overall survival rate of 68.1% at 48 months. However, in the absence of chronic systemic disease, a survival rate of 92% was observed at final follow up. In another prospective study^60^ in 2007 evaluating tantalum implants for the treatment of early osteonecrosis of the femoral head (Stage I or II), 86% of patients demonstrated femoral head survival at a minimum of 2 years follow up and an average follow up of 39 months (range, 27–59 months). Three of 22 had progressive pain and collapse and subsequent conversion to THA. Patients who did not require arthroplasty demonstrated good-to-excellent functional results as characterized by the Harris hip score. Although these data appear promising, long-term follow up is necessary. In addition, there are concerns about the difficulty of conversion to a THA and the generation of metal debris in the joint if arthroplasty becomes necessary.

Meyers^61^ (1978) first reported the application of muscle-pedicle bone graft for the treatment of femoral head ON in 23 patients. With follow-up available between 6 months and 2 years, good results were found in all 8 patients who had Ficat stages I or II disease, but in only 5 of 15 patients who had Ficat stages III or IV disease. Lee and Rehmatullah^62^ reported muscle–pedicle–bone graft for the “silent hip” in idiopathic ON of the femoral head and observed a 70% success rate with their technique. Baksi^63^ (1991) reported his results at 3 to 12 years (mean, 7 years) follow-up in treating 61 patients (68 hips) with a variety of muscle pedicle bone grafts. Of the several types of muscle–pedicle–bone grafts used, the tensor fascia lata anteriorly and the quadratus femoris posteriorly were preferred. As many as 83% of the patients, obtained good or excellent results at follow-up. Stein and colleagues^64^ (2002) reported usage of a muscle pedicle flap for the treatment of ON of the femoral head in 37 patients. Our of which 86% had complete resolution of pain, none had a fixed flexion deformity, and 89% walked without a limp.

Vascularized pedicle bone grafts usually based upon vessels supplying fibula in the form of microvascular pedicles have been found to have undergone minimal creeping substitution and are expected to live on their own vessels to enable quick healing.^5^ There are two types of vascularized grafts (a) free fibular graft using microvascular techniques for harvesting and reimplantation and (b) vascularized iliac crest graft with lesser morbidity, lesser time used as well as technically less demanding surgery.

### Iliac artery pedicled graft

There are three studies for the use of iliac crest vascularised graft. Iwato and colleagues^65^ (1993) reported satisfactory outcome in 17 of the 23 hips (74%) that were treated with deep circumflex iliac artery pedicled iliac crest bone graft. Most patients demonstrated no femoral head collapse preoperatively, but more than 50% had progressed to radiographic collapse at a mean follow-up of 3 years. Pavlovcic *et al*,^66^ reviewed 24 hips with avascular necrosis of the femoral head in 24 patients treated with vascularised iliac bone grafts 12 years after operation. In 7 patients the necrosis was classified as Ficat Stage II and in 17 patients as Stage III. Eight patients showed poor results. In 6 hips with fair results, moderate progression of the necrosis was noted at 3 to 8 years postoperatively. In 5 hips showing good results, slow progression with incipient signs of arthrosis were noted 8 years after the surgery. In the remaining 5 patients with excellent results, no evidence of progression was noted 9 to 14 years postoperatively. The method described is recommended for treatment in the Ficat Stage II and early Stage III, when necrosis does not yet involve the complete femoral head.

Hiroshi *et al*,^67^ (2005) used vascularized pedicle iliac bone graft combined with transtrochanteric anterior rotational osteotomy in patients with extensive necrosis in whom the necrotic area occupied more than two-thirds of the weight-bearing zone of the femoral head. The mean JOA score improved from 67.8 points preoperatively to 78.1 points by 18–133 (mean 50.7) months postoperatively. There was no disease progression to a more advanced stage in 12 of 17 hips (71%) postoperatively. They concluded that vascularized pedicle iliac bone graft combined with transtrochanteric anterior rotational osteotomy to treat avascular necrosis of the femoral head is promising for joint preservation.

### Free vascularized fibular grafting

There are five reports as clinical trials and randomized control studies. Zhang and colleagues^68^ (2000) reported their experience with 48 patients (56 hips) undergoing the free vascular fibular grafting (FVFG) procedure with an average patient age of 37.7 years and an average follow up of 16 months. Etiologies included trauma, steroids, alcohol, and idiopathic. Patients, who had Steinberg^69^ Grade II ON had better Harris hip scores at last follow up than did patients who had Steinberg Stage III or IV disease. The preoperative Harris hip scores for patients who had Steinberg Grades II, III, and IV ONFH were 78.5, 69.3, and 58.4, respectively. At the most recent follow up, the Harris hip scores improved to 94.4, 85.7, and 76.4, respectively.

Marciniak and Shaffer^70^ (2005) reported their series of 101 hips treated with the FVFG procedure with a minimum follow up of 5 years. Sixty-one percent of the hips had not been converted to THA at the 5-year mark and 42% survived until the 8-year postoperative mark. The average Harris hip score for the cohort improved from 58 ± 13 preoperatively to 80 ± 15 at the 5-year mark.

Yoo *et al*^71^ (2008) reviewed 135 patients with 151 hips, who underwent FVFG for osteonecrosis of the femoral head with a follow up of minimum 10 years. The mean Harris hip score improved from 72 to 88. At the latest follow up, they found improved or unchanged radiographs in 37 of 59 Stage II hips and 39 of 65 Stage III hips. Thirteen hips (13 patients, 10.5%) failed treatment and underwent total hip arthroplasty. The location and size of the necrotic lesion and the patient's age influenced long-term survival of the graft. Their data suggested that free vascularized fibular grafting was successful in maintaining joint function and delaying the need for joint replacement procedure. Aldrige *et al*,^72^ (2008) suggested modifications in the FVFG surgical technique, where they suggested confirmation of patency of channels by an intra operative arteriogram after the fibula has been placed within the femoral head but before the anastomosis is completed. This ensured that the pedicle was not kinked, strangulated, or otherwise not flowing properly. They also suggested that by using, computer navigation with standard fluoroscopy and/or CT for exact localization of the necrotic lesion, better accuracy can be expected in the core placement into the necrotic lesion while decreasing the total radiation exposure.

## OSTEOTOMIES

Various osteotomies have been performed to rotate the necrotic or collapsing segment of the hip out of the weight bearing zone, replacing it with a segment of articular cartilage of the femoral head supported by healthy viable bone. In addition to the biomechanical effect, osteotomy may also reduce venous hypertension and decrease intramedullary pressure. Two main types have been described: Trans-trochanteric rotational and Intertrochanteric varus or valgus osteotomy (usually combined with either flexion or extension).

Intertrochanteric osteotomies including both varus and valgus osteotomies, have been reported to give satisfactory results in 73% of cases after a mean follow-up of 5.3 years.^73^ Maistrelli *et al*,^74^ noted that 71% of hips had satisfactory results at two years after intertrochanteric varus or valgus osteotomy and 58% at 8.2 years. Gallinaro and Masse^75^ (2001) described satisfactory results in 62.5% of cases after flexion osteotomy at a mean follow-up of 10.2 years. Flexion valgus osteotomy with autogenous bone-grafting has shown a survival rate of 87% without replacement arthroplasty ten years after operation.^76^ Sugioka's rotational osteotomy is another option for the treatment of osteonecrosis and is designed to move the femoral head into any degree of rotation and varus angulation with the reported success^77^ of 78% after three to 16 years follow-up. Sakano *et al*^78^ (2002) used rotational osteotomy and reported in a success rate of 62% after three to seven year follow-up. They observed that curved varus osteotomy is less invasive and less technically demanding and also a reliable procedure because it increases the intact area in the femoral head on the weight-bearing portion as planned pre-operatively.

Atsumi *et al*,^79^ have (2006) observed that many young patients have extensive lesions with advanced collapse that would not be candidates for the procedure of Sugioka procedure. For these patients, from the remaining anterior area of living femoral head, bone can be moved to the loaded lateral portion of the joint with use of a posterior rotational osteotomy. This operation may delay the progression of degeneration if adequate living bone of the femoral head can be placed under the loaded lateral portion of the acetabulum.

The overall perception is that osteotomies are best suited to patients not being treated with long term steroids, with minimal osteoarthritic changes, with no loss of joint space or acetabular involvement and a small combined necrotic angle.

There are hardly any Indian studies on the subject except a review of using various methodologies in the treatment of AVN by Babhulkar^80^ (2006) in 305 hips of Ficat stage I-III. A total of 160 hips were treated by phemister type of fibular bone grafting, 12 were treated with cancellous bone grafting, 16 with Meyers bone grafting, 10 with Sartorius muscle pedicle grafting, 45 hips with tensor fascia lata pedicle bone and 62 hips with vascularised iliac crest grafting. All these patients were subsequently evaluated for progression to collapse more than 3 mm at the end of 18 months. Radiologically there was progression of disease to collapse in 36% hips after core decompression as against 31% in hips after CD supplemented by bone grafting. Various osteotomies showed collapse in 33-50% cases. TFL muscle pedicle bone grafting procedure and vascularised iliac crest grafting showed collapse in 28.88% and only 16.12% cases respectively. In both these groups a dramatic radiological attempt towards revascularization and remolding was observed. With these observations Babhulkar^80^ suggested that more biological procedures like TFL grafting and vascularised iliac crest grafting add on to the only mechanical effect created by bone graft. Osteotomies show successful results only when performed for localized lesion in segmental necrosis of femoral head and in lesions with minimal collapse of the femoral head.

To conclude early diagnosis and intervention prior to collapse of the femoral head is the key to a successful outcome of joint preserving procedures. New pharmacological measures as well as the use of growth and differentiation factors may eventually alter the treatment outcomes, but it is necessary to await the results of clinical research with long-term follow-up of these patients. The surgical innovations currently under investigation represent modifications of standard core decompression. The preliminary results show that the additional use of bone morphogenic protein and bone marrow stem cells may provide the opportunity to enhance the results of core decompression. In general, patients with collapse of the femoral head will require an arthroplasty procedure.
